# Measurement of wavelength-dependent radiation pressure from photon reflection and absorption due to thin film interference

**DOI:** 10.1038/s41598-018-34381-z

**Published:** 2018-10-29

**Authors:** Dakang Ma, Jeremy N. Munday

**Affiliations:** 1 Department of Electrical and Computer Engineering, University of Maryland, College Park, 20742 USA; 2 Institute for Research in Electronics and Applied Physics, University of Maryland, College Park, 20742 USA

## Abstract

Opto-mechanical forces result from the momentum transfer that occurs during light-matter interactions. One of the most common examples of this phenomenon is the radiation pressure that is exerted on a reflective surface upon photon reflection. For an ideal mirror, the radiation pressure is independent of the wavelength of light and depends only on the incident power. Here we consider a different regime where, for a constant input optical power, wavelength-dependent radiation pressure is observed due to coherent thin film Fabry-Perot interference effects. We perform measurements using a Si microcantilever and utilize an *in-situ* optical transmission technique to determine the local thickness of the cantilever and the light beam’s angle of incidence. Although Si is absorptive in the visible part of the spectrum, by exploiting the Fabry-Perot modes of the cantilever, we can determine whether momentum is transferred via reflection or absorption by tuning the incident wavelength by only ~20 nm. Finally, we demonstrate that the tunable wavelength excitation measurement can be used to separate photothermal effects and radiation pressure.

## Introduction

Radiation pressure was first quantitatively described by Maxwell based on his wave-theory of electromagnetism published in 1873^1^. Nearly thirty years later, radiation pressure was experimentally demonstrated independently by Nichols and Hull^2^ and by Lebedev^3^. In the past decade, there has been renewed interest in radiation pressure due to the development of opto-micro-mechanical devices and the field of cavity optomechanics^4–7^, as well as the practical development of space propulsion techniques using on solar or laser-based sails^8,9^. Radiation pressure has also been considered for use as a calibration method for the cantilever spring constant in atomic force microscopy^10^ and high-power laser power measurements^11^. Endeavors have also been made to explore the effect of radiation pressure in novel systems involving plasmonic materials^12^ and negative-index metamaterials^13–16^. However, even minute amounts of absorption can give rise to photothermal effects, which are difficult to quantify and can obscure measurement of the radiation pressure.

Previous measurements of radiation pressure have been based on a single wavelength excitation^10,17,18^. For a perfectly reflecting mirror, the radiation pressure only depends on the incident optical power and is independent of wavelength. However, for real materials, wavelength-dependent reflection and absorption coefficients will generate different amounts of radiation pressure given the same incident power. The total radiation force per unit incident power is given by:

1 $$\frac{{F}_{rp}}{P}=\frac{2r(\lambda )+a(\lambda )}{c}cos\,\theta ,$$

where *F*_*rp*_ is the photon radiation force, *P* is the incident optical power, *r(λ)* and *a(λ)* are the reflection and absorption coefficients at incident angle *θ*, and *c* is the speed of light.

Observing the wavelength-dependent radiation pressure based on material dispersion is difficult because of the limited variation of reflectivity and absorptivity over the typical measurement bandwidth. However, for a thin film structure, such as a microcantilever, interference effects can cause a dramatic change in the reflectivity and absorption within a short wavelength range. Another challenge in the measurement lies in providing the reflection and absorption coefficients (RHS of Eq. 1) for a microcantilever. These coefficients cannot be calculated from the nominal cantilever thickness, because the thickness is not well controlled during the commercial fabrication process and has large variations even within the same batch^19^. Therefore, *in-situ* measurements of the cantilever’s thickness at the laser excitation position is essential.

In this paper, we demonstrate the first measurement of the wavelength-dependent radiation pressure due to thin film interference in a silicon microcantilever. We show that the cantilever thickness at the excitation position and the incident angle can be determined from the *in-situ* transmission spectrum measurement and nonlinear fitting to the theoretical model. The fitted thickness and incident angle are then used to calculate the radiation pressure and are found to agree with the radiation pressure measured from the cantilever deflection to within the errors associated with the measurement and the calculation. We also show that the tunable wavelength excitation measurement can be used to distinguish the photothermally driven oscillation from the radiation pressure driven oscillation by conducting the same experiment near the base and near the free end of the cantilever, respectively.

**Figure 1:**
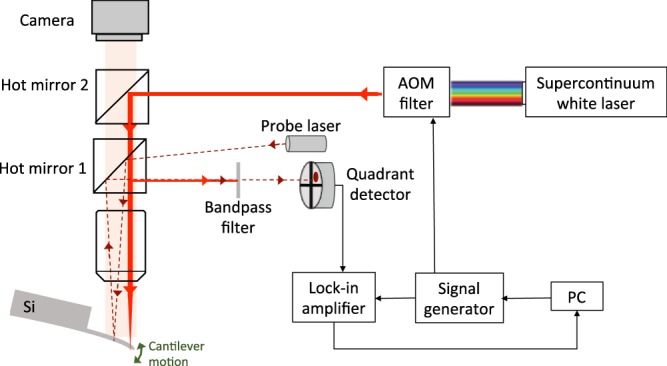
Experimental setup for measuring radiation pressure under tunable-wavelength laser excitation. The thin film interference effect in the Si cantilever causes the cantilever to experience wavelength-dependent radiation pressure and photothermal effects given the same incident optical power for different wavelengths.

The experimental setup for measuring the wavelength-dependent radiation pressure is based on a modified atomic force microscope (Asylum Research, Cypher AFM), see Fig. 1. A supercontinuum “white” laser paired with an acoustic-optic modulator (AOM) filter allows us to tune the output wavelength continuously throughout the visible (see Methods).

**Figure 2:**
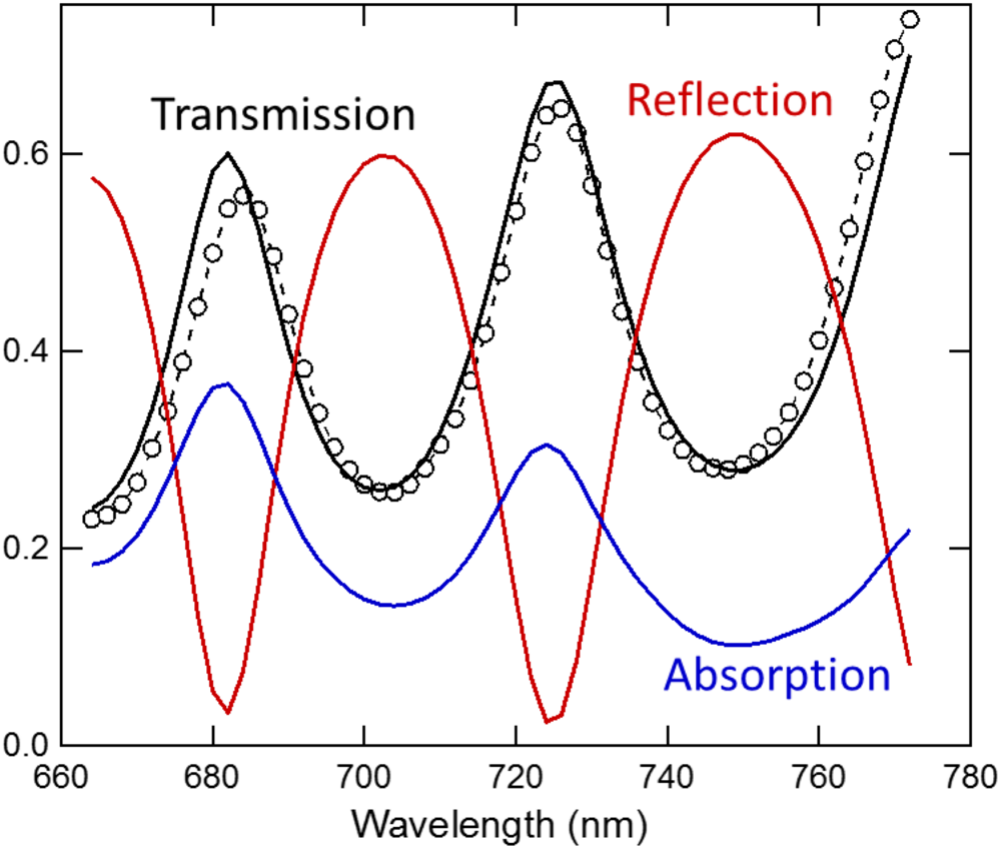
Optical properties of the cantilever at an excitation position near the free end of the cantilever. Black circles represent the measured transmission spectrum (2 nm step size), and the black solid line represents the fitted transmission spectrum based on the thin film interference model. Red and blue solid lines represent the calculated reflection and absorption spectrum from the thickness and incident angle obtained from the transmission spectrum fit.

For the radiation pressure to contribute most effectively to the deflection signal, the excitation laser is focused near the free end of the cantilever (Fig. 1). The weaker probe laser is placed near the middle of the cantilever, away from the excitation laser to avoid Fano-like resonances caused by stray light and local thermal deformation not coupled into cantilever oscillation^20^. The transmission spectrum of the cantilever at the excitation position is measured to determine the local cantilever thickness and incident angle, shown in Fig. 2 (see Methods).

The measured transmission spectrum through the cantilever is used to determine the absorption and reflection from the cantilever. The measured data is fit to the calculated transmission through a single layer of silicon using the transfer matrix method^21^. The two variable parameters in the fitting process are the cantilever thickness and incident angle of the light. Table 1 shows the obtained values for these fit parameters, which are in agreement with the expected range of values. The source is TM polarized, and the index of refraction of Si is obtained from tabulated data^22^. The fitted thickness and incident angle are then used to calculate the reflection and absorption (Fig. 2). Note that the absorption spectrum is peaked when the transmission spectrum is at a maximum, while the reflection spectrum contains minima at these points. These properties can be used to distinguish the dominant driving mechanism of the cantilever. If the measured cantilever amplitude response (normalized by optical power) is opposite of the transmission spectrum, *i.e*. it aligns with the reflection spectrum, the data indicate that radiation pressure excitation is dominant. On the other hand, if the measured cantilever amplitude response aligns with the transmission/absorption spectrum peaks, then the photothermal excitation is usually dominant (note: radiation pressure due to absorption is half the strength of the pressure due to reflection).

**Table 1:** Fitted parameters based on the transmission measurement.

Incident light beam position	Thickness (nm)	Incident Angle
Free End	1265 ± 3	29.7 ± 2.8
Base	1056 ± 1	30.6 ± 1.4

**Figure 3:**
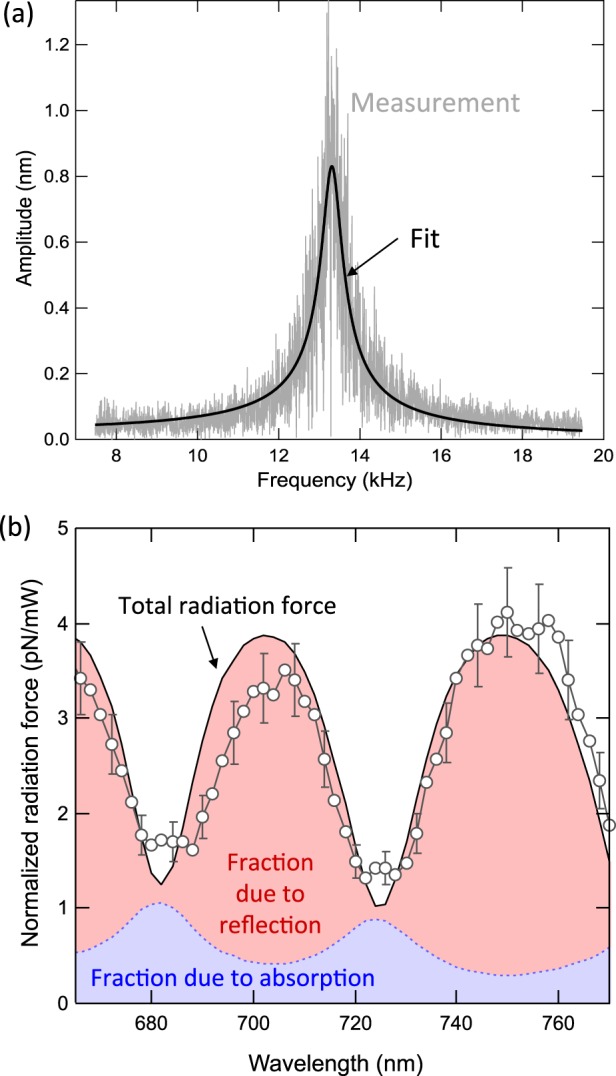
Determination of radiation forces. (**a**) Measured (grey) and fitted (black) amplitude of cantilever oscillation as a function of excitation frequency (tuning curve) at incident wavelength of 750 nm and laser power of 0.37 mW. (**b**) Measured and calculated radiation force normalized by the incident optical power *F*_0_*/P*_0_. The black curve represents the total *F*_0_*/P*_0_ calculated by Eq. (1) using the reflection and absorption spectrum shown in Fig. 2. The red (blue) shaded region shows the proportion of the force that is due to the momentum transfer from photon reflection (absorption). The grey circles represent the measured *F*_*rp*_*/P*_*in*_, and the error bars are plotted every three data points for clarity.

In order for radiation pressure to be the dominant driving mechanism, it is also necessary to focus the external laser beam near the free end. The amplitude of cantilever oscillation under sinusoidal excitation is measured every 2 nm for incident wavelengths between 664 nm and 772 nm. At each wavelength, the laser modulation frequency *ω* is swept across the fundamental resonance frequency of the cantilever, and the cantilever oscillation amplitudes are recorded, forming a tuning curve (Fig. 3a). The tuning curve is described by a simple harmonic oscillator model,

2 $$A(\omega )={A}_{0}\frac{{\omega }_{0}^{2}}{\sqrt{{({\omega }_{0}^{2}-{\omega }^{2})}^{2}+{(\omega {\omega }_{0}/Q)}^{2}}}.$$

The fundamental resonant frequency *ω*_0_ and quality factor *Q* remain the same for all wavelengths. They are obtained from fitting the tuning curve with the largest signal-to-noise ratio (illumination with λ = 750 nm for our setup). The zero-frequency amplitude *A*_0_ is a fitting parameter that describes the magnitude of the oscillation for each illumination wavelength. The measured force for a particular wavelength is calculated as *F*_0_ = *A*_0_*k*_0_, where *k*_0_ is the spring constant of the fundamental frequency calibrated by the thermal method^23^. The measured force (normalized by incident optical power) is determined and compared to the calculated force per incident power based on the measured transmission (Eq. 1); good agreement is found between these values (Fig. 3b). The uncertainty in the measurement comes from the spring constant calibration (±10%), power measurement (±3%), and the fitted incident angle (±9%).

The radiation pressure has two components: one corresponding to momentum transfer from photon reflection and one from momentum transfer upon absorption (Eq. 1). Because the thin film interference condition causes the reflection and absorption to be wavelength-dependent, different incident photon wavelengths lead to different contributions to the radiation pressure based on absorption or reflection. For an incident wavelength near λ = 705 nm, ~90% of the total radiation pressure is due to photon reflection, while at λ = 682 nm, nearly 90% of the total radiation pressure is due to absorption (Fig. 3b). In the case of photon absorption, an additional photothermal bending can exist (see below), but this effect is weak for high frequency modulation of the pump beam incident near the free end of the cantilever^18^.

**Figure 4:**
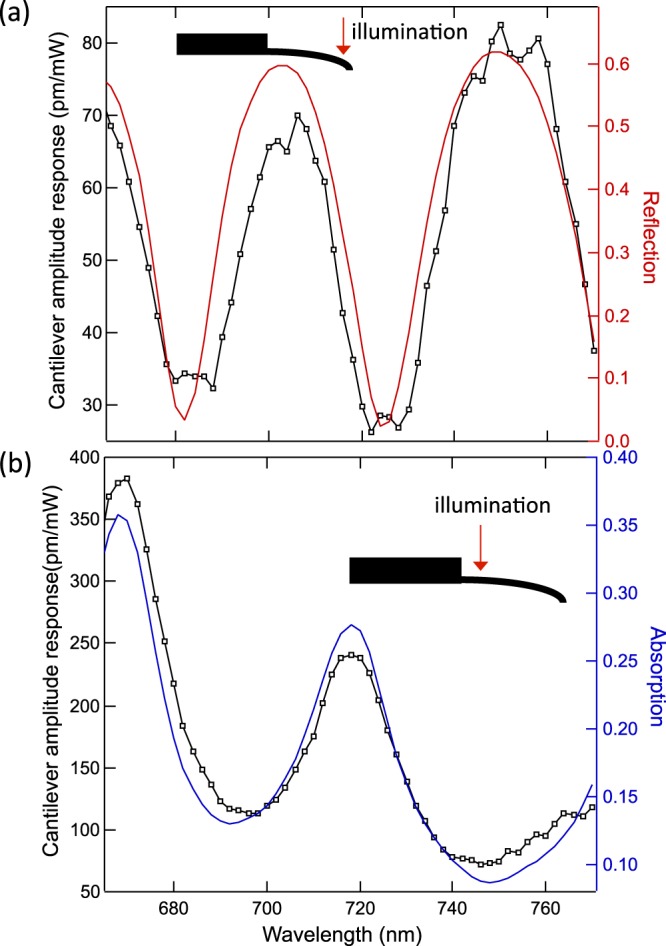
Determination of the dominant driving mechanism for the cantilever oscillation. Cantilever response for laser excitation (**a**) near the free end and (**b**) near the base of the cantilever. Left axis: Cantilever amplitude response (fitted zero-frequency amplitude *A*_0_) normalized by the incident optical power. Right axis: (**a**) reflection and (**b**) absorption. (**a**) For excitation near the free end, the maxima in the amplitude response occur when the reflection is also a maximum, showing a radiation pressure dominated behavior. (**b**) Correlation between absorption maxima and cantilever response for excitation near the base shows behavior is dominated by photothermal effects. The different periodicities shown in the data in (**a**) and (**b**) are caused by the change in cantilever thickness at the excitation positions (free end and base).

The tunable wavelength excitation measurement discussed here also enables a method to determine whether the dominant driving mechanism of the cantilever oscillation is radiation pressure or photothermal when illuminating different regions along the cantilever. To demonstrate this effect, another experiment is conducted with the external laser excitation near the base of the cantilever. The same procedure is used to obtain the absorption spectrum from the measured transmission spectrum (Fig. 4). It is clear that, in the case of excitation near the free end, the normalized cantilever oscillation amplitude aligns with the reflection spectrum (Fig. 4a), indicating that radiation pressure is the dominant driving mechanism. However, for excitation near the base (Fig. 4b), the normalized cantilever oscillation amplitude is proportional to the absorption spectrum, indicating that the driving mechanism is the photothermal bending moment caused by photon absorption^24^. This conclusion is in agreement with our previous results at a single illumination wavelength^18^.

By adjusting the incident wavelength and position of the laser beam on the cantilever, the different components of the radiation pressure and photothermal effects can be tuned. Photothermal effects are maximized for illumination near the base of the cantilever (see also ref.^18^) for wavelengths where absorption is a maximum (*e.g*. near λ = 670 nm and 720 nm). Radiation pressure is greatest for illumination near the free end at wavelengths where reflection is maximized (*e.g*. near λ = 705 nm and 750 nm), because reflection imparts twice as much momentum as absorption (see Eq. 1).

The ability to isolate radiation pressure and photothermal effects using the methods described here may be useful for a number of applications related to optically-induced mechanical motion. In dispersive media, clearly identifying these components is critical to the interpretation of data where the partitioning of momentum between electromagnetic and mechanical components is complicated, such as in experiments related to the Abraham-Minkowski controversy^25,26^. Further, the radiation pressure involving materials with a negative index of refraction becomes more complex upon the addition of absorption, which is an intrinsic feature of these materials^13–16^. Additionally, studies to explore the parameter space over which radiation pressure or photothermal effects dominate the cantilever’s motion and its dependence of geometry and optical and mechanical properties could shed light on applications related to atomic force microscopy (e.g. for driving cantilever oscillations near its resonant frequency) and for studies of the coupling between different modes of excitation.

In summary, we have measured of the wavelength-dependent radiation pressure due to thin film interference in a Si microcantilever over a continuous wavelength range and shown the individual contributions due to photon reflection and absorption, as well as photothermal bending. We developed a technique to obtain the local reflection and absorption spectrum from an *in-situ* transmission measurement by fitting the cantilever thickness and incident angle as an intermediate step. We also showed that the tunable wavelength excitation measurement is a good way to distinguish photothermally driven oscillation (dominant for excitation near the base) from radiation pressure driven oscillation (dominant for free end excitation) through the comparison of the normalized cantilever amplitude spectrum and the reflection or absorption spectrum. Further, while in the radiation pressure dominated regime, we can also tune between a pressure driven by photon reflection or photon absorption by changing the incident wavelength. We expect that the ability to control these interactions will enable further development of opto-mechanical devices and allow for future studies of the coupling between optical and thermal effects in such systems.

## Methods

### Optical excitation of cantilever

The combination of a super continuum white fiber laser (Fianium WhiteLase SC400UV) and an AOM filter serve as the tunable laser source to excite the oscillation of the silicon cantilever. The bandwidth of the AOM filter is 2 nm. The wavelength range used in this experiment is 664 nm to 772 nm. This wavelength window is limited by the output power of the AOM filter (power is too low below 664 nm) and the band-pass filter in front of the quadrant detector used to measure the cantilever deflection (excitation above 772 nm will cause stray light to enter the quadrant photodetector and interfere the probe laser signal).The laser beam is directed and focused on the backside of the cantilever through the same objective (20×) as the probe laser beam (860 nm). The output optical power of the excitation laser is modulated sinusoidally by driving the AOM with a sinusoidal reference signal from a lock-in amplifier. The frequency of the sinusoidal excitation is swept from near dc across the fundamental frequency of the cantilever to amplify the oscillation. The cantilever deflection is detected by monitoring the probe laser beam reflected off the back of the cantilever with a quadrant photodiode, which is then fed into the lock-in amplifier to obtain the frequency response of the amplitude and phase of the oscillation.

### Measurement of cantilever transmission spectrum

An uncoated rectangular cantilever (Mikromasch CSC38) is used in the experiment because silicon has well-known refractive indices across the whole spectrum. An optical power meter (Thorlab PM100D and S130C) is placed underneath the cantilever to measure the optical power transmitted through the cantilever. This transmission spectrum is determined as the ratio of the power measured by a photodetector placed underneath the cantilever to the power measured when the cantilever is removed after the experiment.

## References

[1] Maxwell, J. C. *A Treatise on Electricity and Magnetism*, 1st ed. (Oxford University, 1873).

[2] Nichols E. F., Hull G. F. (1903). The Pressure Due to Radiation. (Second Paper.). Physical Review (Series I).

[3] Lebedev, P. N. *Ann. Phys* **6**, 433 (1901).

[4] Chan, J. *et al*. *Nature* **478**, 89 (2011). 10.1038/nature10461 21979049

[5] Gigan S (2006). Aspelmeyer, and a Zeilinger. Nature.

[6] Kleckner Dustin, Bouwmeester Dirk (2006). Sub-kelvin optical cooling of a micromechanical resonator. Nature.

[7] Schliesser, A. Del’Haye, P. Nooshi, N. Vahala K. J. & Kippenberg T. J. *Phys. Rev. Lett*. **97**, 243905 (2006). 10.1103/PhysRevLett.97.243905 17280288

[8] Ma,D. Murray J. & Munday J. N. *Adv. Opt. Mater*. 1600668 (2016).

[9] Lubin, P. *A Roadmap to Interstellar Flight*, arXiv:**1604**.01356 (2016).

[10] Wilkinson Paul R., Shaw Gordon A., Pratt Jon R. (2013). Determination of a cantilever's mechanical impedance using photon momentum. Applied Physics Letters.

[11] Williams Paul A., Hadler Joshua A., Lee Robert, Maring Frank C., Lehman John H. (2013). Use of radiation pressure for measurement of high-power laser emission. Optics Letters.

[12] Guan, D. *et al*. *Sci. Rep*. **5**, 16216 (2015). 10.1038/srep16216 PMC4653639 26586455

[13] Lezec, H. J. & Chau, K. J. Conf. Lasers Electro-Optics 2009 Conf. Quantum Electron. *Laser Sci. Conf*. **2009** (2009).

[14] Nemirovsky Jonathan, Rechtsman Mikael C., Segev Mordechai (2012). Negative radiation pressure and negative effective refractive index via dielectric birefringence. Optics Express.

[15] Mansuripur, M. & Zakharian, A. R. In *Metamaterials Fundam. Appl. V*, edited by Boardman, A. D., Engheta, N., Noginov, M. A. & Zheludev, N. I., p 845511 (2012).

[16] Chau Kenneth J., Lezec Henri J. (2012). Revisiting the Balazs thought experiment in the case of a left-handed material: electromagnetic-pulse-induced displacement of a dispersive, dissipative negative-index slab. Optics Express.

[17] Marti O., Ruf A., Hipp M., Bielefeldt H., Colchero J., Mlynek J. (1992). Mechanical and thermal effects of laser irradiation on force microscope cantilevers. Ultramicroscopy.

[18] Ma, D. Garrett J. L. & Munday J. N. *Appl. Phys. Lett*. **91107**, 4 (2015).

[19] Salmon, A. R. Capener, M. J. Baumberg J. J. & Elliott S. R. *Meas. Sci. Technol. Meas. Sci. Technol. Meas. Sci. Technol* **25**, 15202 (2014).

[20] Kadri Shahrul, Fujiwara Hideki, Sasaki Keiji (2011). Fano-like resonance in an optically driven atomic force microscope cantilever. Optics Express.

[21] Orfanidis, S. J. *Electromagnetic Waves and Antennas* (Rutgers University, New Brunswick, NJ, 2002).

[22] Palik, E. D. *Handbook of Optical Constants of Solids* (Academic press, 1998).

[23] Cook S M, Lang K M, Chynoweth K M, Wigton M, Simmonds R W, Schäffer T E (2006). Practical implementation of dynamic methods for measuring atomic force microscope cantilever spring constants. Nanotechnology.

[24] Vassalli Massimo, Pini Valerio, Tiribilli Bruno (2010). Role of the driving laser position on atomic force microscopy cantilevers excited by photothermal and radiation pressure effects. Applied Physics Letters.

[25] Kemp, B. A. *J. of Appl. Phys*. **109**, 111101 (2011).

[26] Mário, G. Silveirinha *Phys. Rev. A* **96**, 033831 (2017).

